# Marine Ecosystem Response to the Atlantic Multidecadal Oscillation

**DOI:** 10.1371/journal.pone.0057212

**Published:** 2013-02-27

**Authors:** Martin Edwards, Gregory Beaugrand, Pierre Helaouët, Jürgen Alheit, Stephen Coombs

**Affiliations:** 1 Sir Alister Hardy Foundation for Ocean Science, The Laboratory, Citadel Hill, Plymouth, United Kingdom; 2 Marine Institute, University of Plymouth, Drake Circus, Plymouth, United Kingdom; 3 Centre National de la Recherche Scientifique, Laboratoire d'Océanologie et de Géosciences' UMR LOG CNRS 8187, Station Marine, Université des Sciences et Technologies de Lille 1, Wimereux, France; 4 Baltic Sea Research Institute, University of Rostock, Warnemüende, Germany; 5 Marine Biological Association of the United Kingdom, The Laboratory, Citadel Hill, Plymouth, United Kingdom; University of Vigo, Spain

## Abstract

Against the backdrop of warming of the Northern Hemisphere it has recently been acknowledged that North Atlantic temperature changes undergo considerable variability over multidecadal periods. The leading component of natural low-frequency temperature variability has been termed the Atlantic Multidecadal Oscillation (AMO). Presently, correlative studies on the biological impact of the AMO on marine ecosystems over the duration of a whole AMO cycle (∼60 years) is largely unknown due to the rarity of continuously sustained biological observations at the same time period. To test whether there is multidecadal cyclic behaviour in biological time-series in the North Atlantic we used one of the world's longest continuously sustained marine biological time-series in oceanic waters, long-term fisheries data and historical records over the last century and beyond. Our findings suggest that the AMO is far from a trivial presence against the backdrop of continued temperature warming in the North Atlantic and accounts for the second most important macro-trend in North Atlantic plankton records; responsible for habitat switching (abrupt ecosystem/regime shifts) over multidecadal scales and influences the fortunes of various fisheries over many centuries.

## Introduction

It is well documented that the fortunes of European fisheries such as the Atlantic herring (*Clupea harengus*) and the sardine (*Sardina pilchardus*) fisheries have fluctuated for many hundreds of years, quite often showing alternating successes [1]. It has been hypothesised that climate variation in the form of alternating cyclic periods are responsible for these herring and sardine periods [2]. The impact of natural high frequency (∼7–25 years) atmospheric modes like the North Atlantic Oscillation/Arctic Oscillation (NAO/AO) as well as the impact of climate warming on biological time-series has been widely reported in the literature from both the terrestrial and marine realms [3], [4]. Much of this evidence consists of biological time-series of a few of decades (∼10–30 years) and even shorter-term satellite observations. As a consequence, marine ecosystems responses particularly over the multidecadal scale (>50 years) are still largely unknown. This, coupled with the strong warming trend over the last few decades [5], could lead to the further masking of these natural oscillations and represents a strong bias in reporting biological responses to regional climate warming using short time-series. For example, the warming of the North-east Atlantic experienced over the last few decades has some similarities in terms of both the marine biota present and the hydro-climate of the early 1930s.

The Atlantic Multidecadal Oscillation (AMO) with its alternating warm and cool phases has consequences for large parts of the Northern Hemisphere from modulating El Nino-Southern Oscillation teleconnections as well as many other climate related phenomena such as Atlantic hurricane formation, African drought frequency and winter temperatures in Europe [6]–[8]. While instrumented observations are only capable of revealing two full cycles of the AMO, tree-ring based reconstructions, ice-core records and model simulations have revealed this oscillation occurring at low-frequencies (∼60–80 years) for at least 500 years [7], [9], [10]. Although at present the dynamics of the oscillation itself is somewhat unknown, it has been hypothesised that it arises primarily from changes in the meridional overturning circulation [8].

## Results

**Figure 1:**
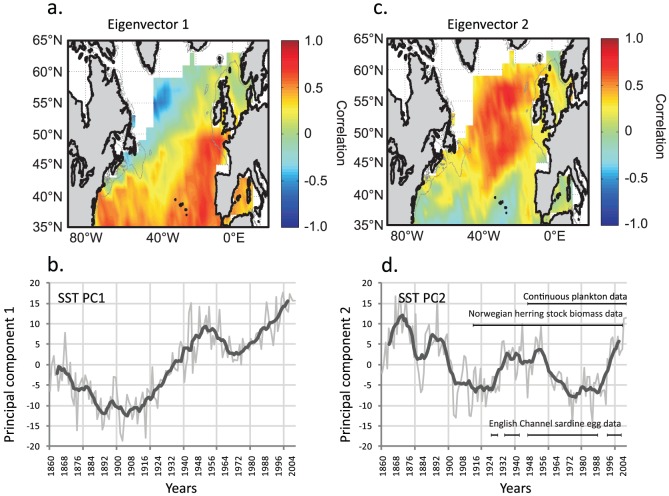
Long-term changes in Sea Surface Temperature in the North Atlantic since 1860. **a**. Spatial distribution of eigenvector 1. **b**. First Principal Component time-series corresponding with eigenvector 1. **c**. Spatial distribution of eigenvector 2. **d**. Second Principal Component time-series corresponding with eigenvector 2. Marine biological time-series used in the biological analysis are superimposed on Figure 1.d. Light lines: annual means, dark lines: 5 year running means.

To examine the relationship between marine ecosystem changes and the long-term changes in Sea Surface Temperature (SST) we first isolated the multi-decadal oscillatory behaviour of the North Atlantic SST (reflecting changes in the AMO and hereinafter referred to as the AMO signal) by performing a standardised Principal Component Analysis (PCA) on long-term spatial changes in SST over the period 1860–2006. While there are a number of methods to isolate the AMO signal from the SST records, the main purpose no matter the methodology employed is to isolate the oscillatory behaviour of the SST pattern [5]–[8]. We did not need to remove the linear trend in North Atlantic SST observations as it was identified in the first principal component and therefore did not appear in the second component. The resulting eigenvector maps (indicating the correlations in space of SST with the corresponding principal component) and the time-series associated with each eigenvector known here as Principal Component Time-Series (PC-TS) are shown in Fig. 1. The first PC-TS (SST PC1; 16.74% of the total variance) was found to be dominated by the Northern Hemisphere temperature trend (r = 0.83; p_ACF_ = 0.02) from 1860–2006 (Fig. 1b). The oscillatory North Atlantic temperature trend (SST PC2) was the second most dominant signal in North Atlantic temperature records explaining 10.38% of the total variance (Fig. 1d). The second component was found to be most spatially prevalent in the central oceanic waters of the North Atlantic (Fig. 1c). Over the instrumented records (1856 to present) alternating warm and cool periods can be seen exhibiting a ∼60–80 year cycle (i.e. the AMO signal). Warm phases are evident between 1860–1890 and 1930–1960 with another warm phase occurring after 1995. Cool phases occur between 1905–1925 and 1970–1990 (Fig. 1d). Therefore, the AMO can alternately mask and enhance the increase in SST in the Northern Hemisphere.

**Figure 2:**
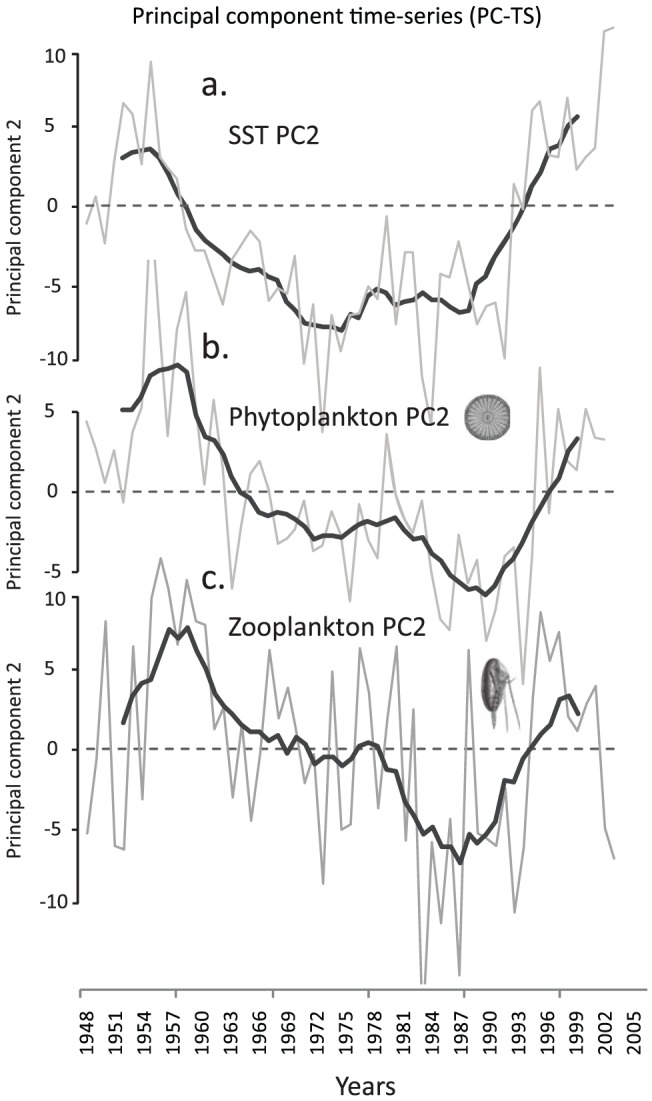
Long-term changes in the plankton of the North Atlantic since 1948 represented by the second most important trend. **a**. Second Principal Component time-series of SST taken from Fig. 1.d but shortened to 1948. **b**. Second Principal Component time-series of the Phytoplankton Colour Index (representing total phytoplankton standing stock). **c**. Second Principal Component time-series of *Calanus* spp. abundance (inverted); a dominant copepod genus of the North Atlantic and representing zooplankton. Light lines: annual means, dark lines: 5 year running means.

To explore the main trends of plankton for the period 1948-present we used the Phytoplankton Colour Index (an index of phytoplankton biomass) and the abundance of *Calanus* spp. (the dominant zooplankton copepod of the North Atlantic) recorded by the Continuous Plankton Recorder (CPR) survey and applied the same technique described for the temperature records. The resulting eigenvector maps and PC-TS revealed the dominant trend to be associated with the SST PC1 accounting for 39.86% of the variance for phytoplankton (showing a general increase) and 14.95% for zooplankton (showing a decrease in abundance associated with the boreal species *Calanus finmarchicus*) (See Figures S1 & S2). The second dominant trend explained 12.89% and 11.28% of the total variance for both the phytoplankton (PC2) and zooplankton (PC2), respectively (Fig. 2). They were both associated with SST PC2 (i.e. the AMO signal, data from Fig. 1d and shown in Fig. 2a). Inter-annual correlations between the second principal component resulting from the PCA on SST and *Calanus* spp. was r = 0.33 (p_ACF_<0.05) and correlation between the same principal component and PCI (PC2) was r = −0.53 (p_ACF_<0.05). Although the inter-annual correlations show considerable variability and are fairly weak at the ocean basin-scale due to the heterogeneous spatial nature of the AMO, the multi-decadal trend clearly dominates the time-series. This biological signal was most spatially prevalent in oceanic waters to the north west of the British Isles and south of Iceland (where it represents the dominant trend in both SST and phytoplankton, see Figures S1 & S2). The CPR data appear to catch the tail end of the last warm AMO phase (∼early 1960s) before declining over many decades until switching along with the AMO signal after 1995 (Fig. 2).

**Figure 3:**
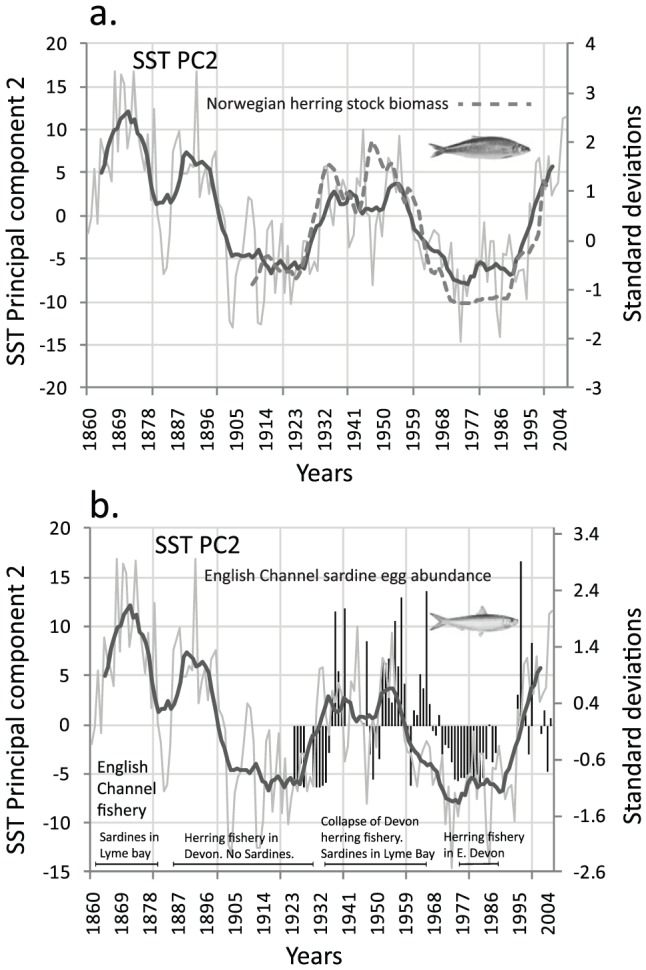
Long-term changes in fish abundance in the North Atlantic. **a**. Second Principal Component time-series of SST taken from Fig. 1.d and Norwegian herring stock biomass (dashed line) between 1908–2000, data based on (12). Norwegian stock biomass values standardised to zero mean and unit variance. **b**. Second Principal Component time-series of SST taken from Fig. 1.d and sardine egg abundance (bars) from the English Channel from 1924–2004. Sardine egg abundance values standardised to zero mean and unit variance (bars used as time-series is not continuous). Fluctuations in the fisheries of the English Channel superimposed on Fig. 3.b based on [1]. Post 1995 dominated by sardines in Western English Channel.

In contrast to Drinkwater [11], who described multidecadal climate changes in response to the distribution and population dynamics mainly of ground fish and benthos in the Arctic between 1920–1940, we have focussed on the pelagic realm in more Northern European waters. In examining longer records we have focused on fisheries data and records concerning sardine (*Sardina pilchardus*) egg abundance recorded in the western English Channel (discontinuous records from 1924–2004) and Norwegian Atlantic Herring stock biomass (*Clupea harengus*, data from 1908–2000). The reason being is that both these datasets date back to the early 20^th^ century with this combined with well documented historical records dating back to the 16^th^ century for the fluctuating herring and sardine fishery based in the English Channel [1] (fluctuations in the fisheries superimposed on Fig. 3b back to 1860). The trends for both herring and sardine show similar multi-decadal trends to the AMO signal (Fig. 3). The correlations between the second principal component reflecting the AMO signal and both species were significantly positive (sardine: r = 0.49; p<0.01; herring: r = 0.91; p_ACF_<0.01). Although the mechanism contributing to the phase lag observed between the temperature and sardine egg abundance cannot be entirely explained. As for most fish species, egg numbers of sardine are directly related to stock size. Stock size responds to changing environmental conditions with a lag of several years, reflecting the age at first maturity (1–2 years) and population age structure (median age 6 years) of sardine in the western English Channel. The English Channel is close to a biogeographic boundary between these two species with the warm-temperate sardine species at its northern extent and the cold-boreal herring species towards its southern extent. Warming periods associated with the AMO therefore favour the sardine population and, conversely, cold periods favour the herring population (Fig. 3b). Further north in the Norwegian and Barents Sea the herring population is close to its most northerly extent with warming in this case favouring the herring populations (Fig. 3a).

## Discussion

The period of warming (warm AMO phase) from the 1930s to the 1960s had profound impacts for the fisheries of the North Atlantic from temperate shelf systems such as the North Sea to boreal and Arctic systems. The warming period caused large poleward distribution extensions of numerous marine and terrestrial species from Greenland to Iceland [11]. For example, the distribution of cod extended poleward along the coast of west Greenland by ∼1000 km during this warming period and the biomass of Norwegian spring-spawning herring increased almost ten-fold [12]. Simultaneously, herring at their southerly distribution in the English Channel collapsed in the early 1930s to be replaced by sardines some years later as the dominant pelagic species [1]. Conversely, when the Norwegian Sea underwent fairly rapid cooling after the late 1950s to the 1970s the herring population declined by more than four orders of magnitude (Fig. 3a). Since the mid 1990s warming event the herring spawning stock has subsequently recovered to levels similar to the mid 1960s (Fig. 3a). While acknowledging the potential contribution from recruitment overfishing [13] the underlying evidence suggests that a relatively small change in basin-scale average temperature (higher in certain regions) can trigger habitat switching where vulnerable populations are found at the extremities of their biogeographic ranges whether this is at the northern or southern limits of their distribution. The large changes in abundance and shifts in fish and plankton species observed in the English Channel between the periods 1920s and 1970s have previously been termed ‘The Russell Cycle’ [14], [15]. Therefore, it appears that what has been termed the Russell Cycle in the English Channel is largely synchronous with the low frequency cyclical variability in North Atlantic SST known as the AMO signal (Fig. 3b).

The abrupt ecosystem shift that occurred in the early 1930s (beginning of a warm AMO phase) was also an exceptional period for the North Sea. This period was marked by the presence of Mediterranean water in the Faroe-Shetland Channel and anomalously high salinities in the southern North Sea in the autumn of 1933. This was coupled with hydro-biological evidence for a major inflow event from fishery incursions into the Baltic from the North Sea by horse mackerel (1932) and anchovy (1933) as well as a dramatic rise in the anchovy stock in the southern North Sea between the period 1930–1934 and the occurrence of unusual warm-water fish species in the North Sea. This warm-water biota present in the North Sea during the 1930s was very similar to the more recently documented regime shift for the North Sea occurring in the mid- to late 1980s [16]–[18].

The major alternation back to the second warm AMO state of the 20^th^ century occurred after 1995 following a 40 year decline in SST with a simultaneous switch in the plankton (Fig. 2) and fish (Fig. 3) as well as numerous other components of the plankton community particularly the microzooplankton and a threefold increase in blue whiting stocks [19]. The ecosystem shift was most prevalent in oceanic waters to the west and north of the British Isles occurring ∼10 years later than the documented North Sea regime shift with the Phytoplankton Colour Index mirroring the major shifts in SST anomalies in both ecosystems. The reasons for these differences in timing of the major shifts are climatically complex but may involve the movement of a critical thermal boundary northwards [20] as well as the different spatial influences of the NAO and the AMO in shallow shelf sea and deep oceanic systems [21]. Another oceanographic and climatic manifestation of the AMO may involve the contraction (warm AMO phase) and expansion of the sub-polar gyre (cold AMO phase). During the peak of the last cold AMO phase (mid to late1970s, a North Atlantic cooling phase also coincident with the Great Salinity Anomaly) the hydro-biological evidence suggests that the sub-polar gyre was positioned much further to the east in the North Atlantic effectively, reducing and capping off the volume of warm North Atlantic water from travelling northwards and from entering the North Sea [22]. Conversely, the period after 1995 (warm AMO phase) was associated with a weaker and a more westerly positioned sub-polar gyre front [23] allowing warmer and more saline water to expand into the Icelandic basin and further north. The redistribution of this warm water further north is coincident with the spatial movement northwards and eastwards of the main centres of action of the NAO and highlights the interplay between the atmosphere and ocean with consequent recent accelerated warming of the Arctic [24].

In conclusion, we have found that the AMO's impact on marine ecosystems has been significant in the past with it influencing a number of biological trends. Firstly, the AMO accounts for the second most important macro-trend in North Atlantic plankton records as a whole and the dominant trend in the oceanic waters to the west and north of the British Isles and the Norwegian Sea. Secondly, the AMO has been responsible for temperature-mediated habitat switching (abrupt ecosystem/regime shifts) in certain regions of the North Atlantic and explains the mechanism for the Russell Cycle in the English Channel. Thirdly, the fortunes of various fisheries stocks in the North Atlantic over a multidecadal scale have been influenced by trends in the AMO. While the two AMO warming periods of the 20^th^ century may at first seem identical in their hydro-biological impact, there is now a fundamental difference between the two periods with the current warming phase increasingly influenced by the monotonic trend in the Northern Hemisphere Temperatures (Fig. 1a). The redistribution of warm Atlantic water further northward post 1995 coupled with the NHT trend is coincident with the rapid climate warming of the Arctic seen over the same period. A fundamental question that then arises, but still remains elusive, is when will the current warm phase of the AMO begin to decline (∼2025 based on 60 year cycle) [25] and will it be significant enough to trigger habitat switching in the North Atlantic and associated shelf seas, or will external climate warming override this natural signal?

## Materials and Methods

Biological data (1948–2006) are taken from the Continuous Plankton Recorder (CPR) survey [26]. We used the Phytoplankton Colour Index (PCI) as a CPR-derived index of phytoplankton standing stock in the marine environment [20] and *Calanus* spp. (representing zooplankton and generally dominated by the species *C. finmarchicus*) abundance. Biological data were interpolated on a grid of 2° longitude×2° latitude from 31°N to 65°N and from 99°W to 11°E using an inverse squared distance interpolation method [27]. The sardine egg abundance estimates (1924–2004) were obtained from quasi-monthly plankton sampling based on an offshore station from Plymouth, see [28] for technical details on methodology. Northern herring stock biomass data based on [12].

Mean annual Sea Surface Temperature (SST; 1860–2006) in the North Sea originated from the International Comprehensive Ocean-Atmosphere Data Set (ICOADS, 2-degree enhanced data). The Atlantic Multidecadal Oscillation (AMO; 1860–2006) is a large-scale oceanic phenomenon, source of a natural variability in the range of 0.4°C in the region covered by this study [8]. Northern Hemisphere Temperature (NHT; 1860–2006) anomalies were used as an index of large-scale change in surface temperature. The data were provided by the Hadley Centre for Climate Prediction and Research, Meteorological Office, Exeter, UK [29].

We used a standardized Principal Component Analysis (PCA); to describe long-term changes in sea surface temperature from 1860 to 2006 over the North Atlantic sector (from 31°N to 65°N and from 99°W to 11°E) described in Beaugrand *et al.* [30]. Two other standardized PCAs were performed on PCI and *Calanus* spp. abundance in the North-east Atlantic (from 40°N to 65°N and from 30°W to 10°E) from 1948 to 2006 to reveal the main long-term changes in these plankton groups.

Correlation analyses were applied between indices of large-scale hydroclimatic variability, long-term changes in SST, PCI and *Calanus* spp. as represented by principal components. The temporal autocorrelation function was calculated to allow an adjustment of the actual degree of freedom.

## Supporting Information

Figure S1 **Long-term changes in** ***Calanus*** **spp abundance in the North East Atlantic.** **a**. Spatial distribution of eigenvector 1 and First Principal Component time-series corresponding with eigenvector 1 for *Calanus* spp. abundance in the North East Atlantic from 1948. **b**. Spatial distribution of eigenvector 2 for *Calanus* spp. abundance in the North East Atlantic from 1948 and Second Principal Component time-series corresponding with eigenvector 2.
(PDF)

Figure S2 **Long-term changes in Phytoplankton Colour in the North East Atlantic.** **a**. Spatial distribution of eigenvector 1 and First Principal Component time-series corresponding with eigenvector 1 for phytoplankton colour in the North East Atlantic from 1948. **b**. Spatial distribution of eigenvector 2 for phytoplankton colour in the North East Atlantic from 1948 and Second Principal Component time-series corresponding with eigenvector 2.
(PDF)
