# Supplementary material for: Marine Ecosystem Response to the Atlantic Multidecadal Oscillation
Source: PLoS One. 2013 Feb 27;8(2):e57212. doi: 10.1371/journal.pone.0057212 (PMC3584106; doi:10.1371/journal.pone.0057212)
Supplement: Figure S1 — Long-term changes in Calanus spp abundance in the North East Atlantic. a. Spatial distribution of eigenvector 1 and First Principal Component time-series corresponding with eigenvector 1 for Calanus spp. abundance in the North East Atlantic from 1948. b. Spatial distribution of eigenvector 2 for Calanus spp. abundance in the North East Atlantic from 1948 and Second Principal Component time-series corresponding with eigenvector 2. (PDF) [file pone.0057212.s001.pdf]

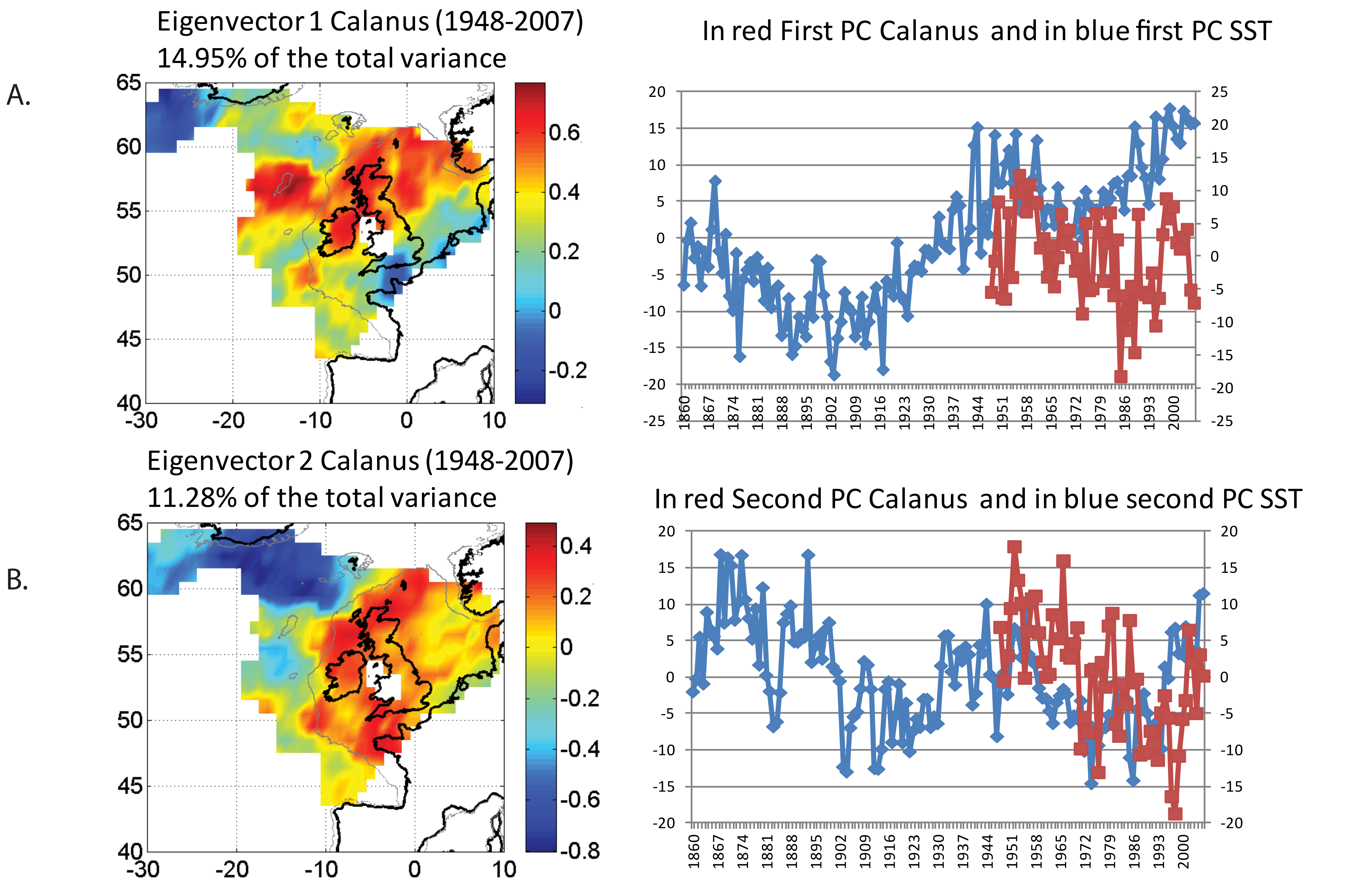
A.
Eigenvector 1 Calanus (1948-2007)
14.95% of the total variance
In red First PC Calanus and in blue first PC SST
B.
Eigenvector 2 Calanus (1948-2007)
11.28% of the total variance
In red Second PC Calanus and in blue second PC SST
65
60
55
50
45
40
-30
-20
-10
0
10
0.6
0.4
0.2
-0.2
0.2
-0.4
-0.6
-0.8
20
15
10
5
-5
-10
-15
-20
-25
25
1860
1867
1874
1881
1888
1895
1902
1909
1916
1923
1930
1937
1944
1951
1958
1965
1972
1979
1986
1993
2000
