# Supplementary material for: Marine Ecosystem Response to the Atlantic Multidecadal Oscillation
Source: PLoS One. 2013 Feb 27;8(2):e57212. doi: 10.1371/journal.pone.0057212 (PMC3584106; doi:10.1371/journal.pone.0057212)
Supplement: Figure S2 — Long-term changes in Phytoplankton Colour in the North East Atlantic. a. Spatial distribution of eigenvector 1 and First Principal Component time-series corresponding with eigenvector 1 for phytoplankton colour in the North East Atlantic from 1948. b. Spatial distribution of eigenvector 2 for phytoplankton colour in the North East Atlantic from 1948 and Second Principal Component time-series corresponding with eigenvector 2. (PDF) [file pone.0057212.s002.pdf]

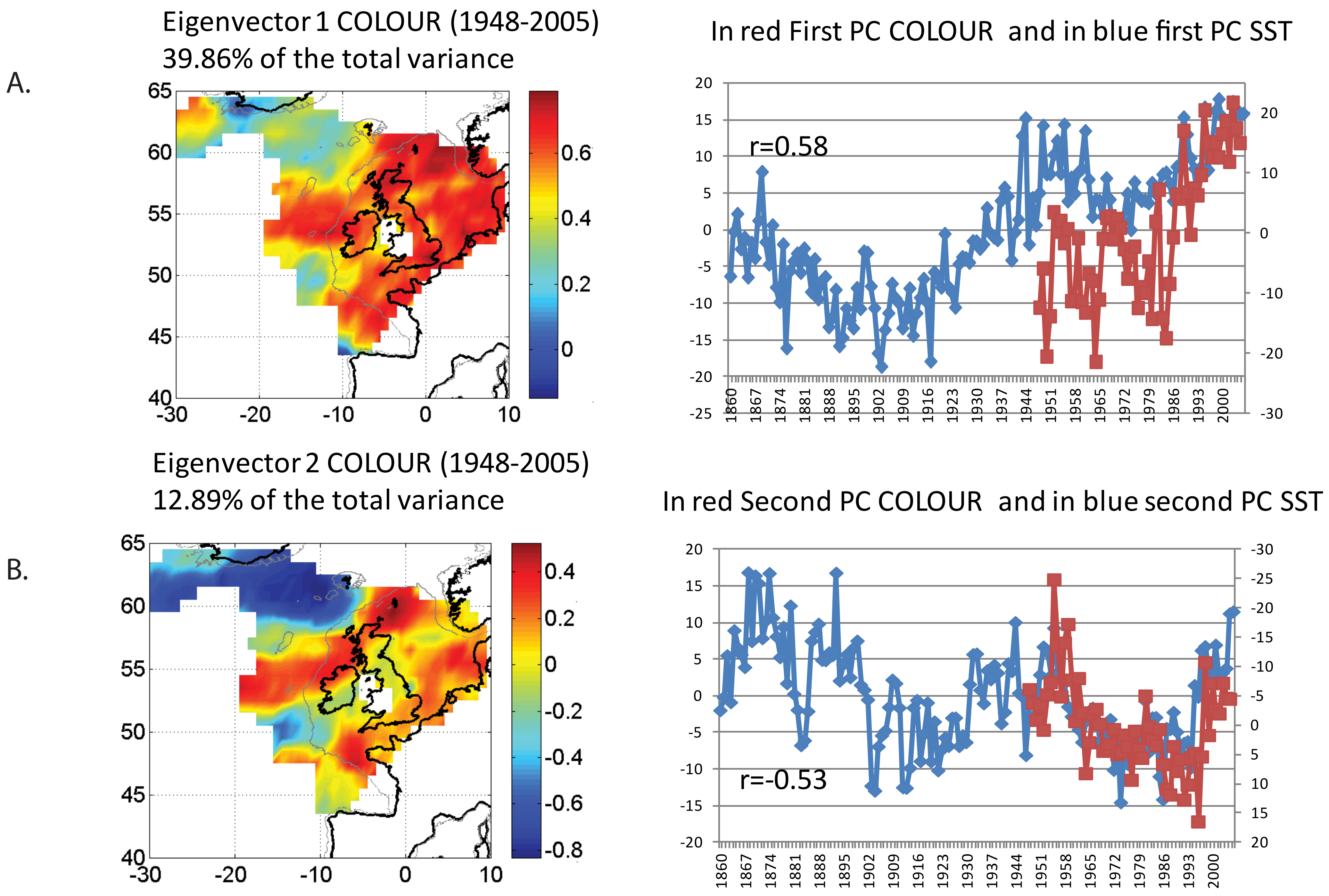
A.
Eigenvector 1 COLOUR (1948-2005)
39.86% of the total variance
In red First PC COLOUR and in blue first PC SST
r=0.58
B.
Eigenvector 2 COLOUR (1948-2005)
12.89% of the total variance
In red Second PC COLOUR and in blue second PC SST
r=-0.53
